# Human milk feeding supports adequate growth in infants ≤ 1250 grams birth weight

**DOI:** 10.1186/1756-0500-6-459

**Published:** 2013-11-13

**Authors:** Amy B Hair, Keli M Hawthorne, Katherine E Chetta, Steven A Abrams

**Affiliations:** 1 Department of Pediatrics, Section of Neonatology, USDA/ARS Children’s Nutrition Research Center, Baylor College of Medicine, Texas Children’s Hospital, Houston, TX, USA

**Keywords:** Neonate, Growth, Nutrition, Human milk, Growth failure, Necrotizing enterocolitis

## Abstract

**Background:**

Despite current nutritional strategies, premature infants remain at high risk for extrauterine growth restriction. The use of an exclusive human milk-based diet is associated with decreased incidence of necrotizing enterocolitis (NEC), but concerns exist about infants achieving adequate growth. The objective of this study was to evaluate growth velocities and incidence of extrauterine growth restriction in infants ≤ 1250 grams (g) birth weight (BW) receiving an exclusive human milk-based diet with early and rapid advancement of fortification using a donor human milk derived fortifier.

**Methods:**

In a single center, prospective observational cohort study, preterm infants weighing ≤ 1250 g BW were fed an exclusive human milk-based diet until 34 weeks postmenstrual age. Human milk fortification with donor human milk derived fortifier was started at 60 mL/kg/d and advanced to provide 6 to 8 additional kilocalories per ounce (or 0.21 to 0.28 kilocalories per gram). Data for growth were compared to historical growth standards and previous human milk-fed cohorts.

**Results:**

We consecutively evaluated 104 infants with mean gestational age of 27.6 ± 2.0 weeks and BW of 913 ± 181 g (mean ± standard deviation). Weight gain was 24.8 ± 5.4 g/kg/day with length 0.99 ± 0.23 cm/week and head circumference 0.72 ± 0.14 cm/week. There were 3 medical NEC cases and 1 surgical NEC case. 22 infants (21%) were small for gestational age at birth. Overall, 45 infants (43%) had extrauterine growth restriction. Weight velocity was affected by day of fortification (p = 0.005) and day of full feeds (p = 0.02). Our cohort had significantly greater growth in weight and length compared to previous entirely human milk-fed cohorts.

**Conclusions:**

A feeding protocol for infants ≤ 1250 g BW providing an exclusive human milk-based diet with early and rapid advancement of fortification leads to growth meeting targeted standards with a low rate of extrauterine growth restriction. Consistent nutritional policies using this approach may be considered for this population.

## Background

Human milk feeding is associated with substantial benefits to the health and development of premature infants [1]. The use of an exclusive human milk-based diet for very low birth weight (VLBW) infants as an alternative to bovine-based fortification of mother’s own milk or bovine-based formula has risen dramatically. An exclusive human milk-based diet is defined as mother’s own milk supplemented with pasteurized donor human milk (when mother’s milk is unavailable) fortified with a donor human milk derived fortifier [2]. Sullivan et al [2] found that an exclusive human milk-based diet in infants ≤ 1250 grams (g) birth weight (BW) reduced the incidence of necrotizing enterocolitis (NEC) by 50% and the rate of surgical NEC by 90%. However, growth failure remains a common concern in exclusively human milk fed VLBW infants. Some studies have shown that donor human milk is associated with slower growth in the early postnatal period [3,4] however; these studies were limited to unfortified donor human milk or donor human milk with bovine milk-based fortifier. The American Academy of Pediatrics policy statement on breastfeeding and the use of human milk recommends that all preterm infants receive human milk including donor human milk if mother’s own milk is unavailable [5].

Extremely premature infants are at risk for slow growth, metabolic abnormalities, and poor neurodevelopmental outcomes [6-8]. Postnatal growth standards are based on estimated intrauterine growth from historical cohort studies and post-mortem analyses [4,9]. Despite current strategies aimed at appropriate nutrition, a large proportion of these infants have postnatal growth failure, also called extrauterine growth restriction (anthropometric values < 10^th^ percentile) [8,10,11]. A cohort study of infants < 28 weeks gestational age showed that growth velocities greater than current guidelines are needed to ensure that infants maintain their birth weight percentile on the growth curve and avoid extrauterine growth restriction [12]. Recommended enteral nutrient intakes per Tsang guidelines [13] for VLBW infants are 110–130 kilocalories per kilogram per day (kcal/kg/d) with 3.4-4.2 grams per kilogram per day (g/kg/d) of protein. For extremely low birth weight infants, recommended enteral nutrient intakes are 130–150 kcal/kg/d with 3.8-4.4 g/kg/d of protein [13]. The ESPGHAN Committee on Nutrition recommends 110–135 kcal/kg/d with 4.0-4.5 g/kg/d of protein for preterm infants receiving enteral nutrition [14]. Adequate growth is key to ensuring improved neurodevelopmental and other outcomes. For infants < 1000 g BW, Ehrenkranz et al [15] found that as the rate of weight gain and head circumference (HC) increased, there was decreased incidence of cerebral palsy and poor neurodevelopmental scores at 18 to 22 months’ corrected age.

Because of the potential benefits of using an exclusive human milk-based diet, but remaining uncertainty about growth, we chose to evaluate the growth in all of our infants ≤ 1250 g BW receiving this diet. In this single center, prospective observational cohort study, we hypothesized that a feeding protocol providing an exclusive human milk-based diet with early and rapid advancement of fortification using a donor human milk derived fortifier would meet growth standards in infants ≤ 1250 g BW and lead to decreased extrauterine growth restriction.

## Methods

Infants were consecutively followed in this single center prospective cohort study from August 2010 to December 2011. Inclusion criteria were: premature infants <37 weeks gestation, BW ≤ 1250 g, admitted within 48 hours of birth, receiving an exclusive human milk-based diet, and achievement of full enteral feedings by 4 weeks of age. Infants were excluded who died within the first week of life and those who had major congenital anomalies. Infants were followed from birth until discharge and data were prospectively collected for growth and nutrition using pre-study defined variables and definitions.

As approved by the Institutional Review Board of Baylor College of Medicine and Affiliated Hospitals, consent was waived for this observational study. It was registered with clinicaltrials.gov (reg. # NCT01204983). Our primary outcome was to evaluate the nutritional management including growth analysis for infants receiving an exclusive human milk-based diet with a donor human milk derived fortifier.

### Standardized feeding protocol

Infants were fed a standardized feeding protocol as tolerated providing an exclusive human milk-based diet. The protocol was maintained throughout the study period and was under twice weekly guidance by a neonatal dietitian. Infants received mother’s own milk supplemented with pasteurized donor human milk when mother’s milk was unavailable. Donor human milk was obtained from a member milk bank of the Human Milk Banking Association of America. Our hospital has a human milk preparation area with dedicated milk bank technicians that follow evidence-based protocols and American Dietetic Association standards specific to milk collection, transportation, storage, preparation, and fortification.

Enteral feeding was started within the first 24 hours of life if medically feasible with trophic feeds of 20 milliliters per kilogram per day (mL/kg/d) for 3 days. While enteral feeding was advanced, goal parenteral nutrition intake as per protocol provided 110 kcal/kg/d with 3.5 g/kg/d of protein and 3 g/kg/d of fat as Intralipid 20% (Baxter). Intralipid was discontinued once feeds were at 80 ml/kg/d and parenteral nutrition was discontinued once volume fell below 50 ml/kg/d or feeds were at 100 to 120 ml/kg/d. Feeds were advanced by 20 mL/kg/d to reach a goal of 140 to 150 mL/kg/d. Human milk was fortified with pasteurized donor human milk derived fortifier, Prolact+H^2^MF® (Prolacta Bioscience, Industry, CA [16]). Human milk fortification occurred at 60 ml/kg/d (HMF 60) to provide an additional 4 kilocalories per ounce (kcal/oz) which is equal to an additional 0.14 kilocalories per gram (kcal/g) and then an additional 6 kcal/oz (0.21 kcal/g) at 100 to 120 ml/kg/day. Assuming infants received human milk at 20 kcal/oz (0.67 kcal/g) with 0.9 g/dL of protein based on reference values for mature milk [17] and full feeds at a volume of 150 ml/kg/day with fortification with a donor human milk derived fortifier of an additional 6 kcal/oz (0.21 kcal/g), then infants received a total of 130 kcal/kg/day with 3.6 g/kg/day of protein. Once full enteral feedings were established, if weight gain did not reach 15 g/kg/d within a week, as per protocol, fortification was advanced to provide an additional 8 kcal/oz (0.28 kcal/g) or 140 kcal/kg/day and 4.4 g/kg/day of protein. The maximum amount of fortification provided to infants was an extra 10 kcal/oz (0.35 kcal/g) or 150 kcal/kg/day and 5.25 g/kg/day of protein. Infants received this diet until approximately 34 weeks postmenstrual age (PMA) and were then transitioned over a period of five days to a specialized formula or bovine milk-based fortifier.

### Data collection

Weight was recorded daily. Length and HC were recorded weekly. Bedside nurses obtained weight using digital baby scales or Giraffe® OmniBed scales (GE Healthcare, Finland). Measurements for length and HC were obtained using a length board and tape measure, respectively. Growth velocities from birth to discharge, were compared to pre-study defined growth standards which were weight gain 15 g/kg/day, length 1.0 centimeters per week (cm/week), and HC 0.7 cm/week based on historical cohort studies [4,9]. Birth and discharge or 40 weeks PMA (if discharged after 40 weeks PMA) anthropometric data were compared to intrauterine-based growth curves published by Olsen et al [18].

Study outcomes included growth velocity (weight, length and HC), number of days to full feeds (tolerating fortified feeds of 140 ml/kg/d), total parenteral nutrition (TPN) days, NEC, spontaneous intestinal perforation, late onset sepsis, ileus associated with sepsis, patent ductus arteriosus, intraventricular hemorrhage (IVH), bronchopulmonary dysplasia, length of stay, discharge weight and number of infants small for gestational age (SGA) at discharge or 40 weeks PMA. We defined NEC as Stage II NEC or greater using modified Bell’s criteria [19]. The presence of pneumatosis on abdominal radiograph was read by a pediatric radiologist. Medical NEC was defined as Bell’s Stage II disease or greater while surgical NEC was defined as NEC requiring surgery during the acute care period. Late onset sepsis was defined as culture positive sepsis after 3 days of age associated with clinical symptoms of sepsis. IVH on head ultrasound imaging was classified per Papile et al. with severe IVH defined as grade III or grade IV [20]. Bronchopulmonary dysplasia was defined as oxygen dependence at 36 weeks PMA. Small for gestational age (SGA) was defined as a weight < 10^th^ percentile on the Olsen growth curve [18]. Appropriate for gestational age (AGA) was defined as a weight ≥ 10^th^ percentile but < 90^th^ percentile on the Olsen et al. growth data [18].

Our cohort data were then compared to previous human milk fed cohorts. Our cohort received a feeding protocol that rapidly advanced human milk fortification as compared to the fortification strategy used in the Sullivan et al [2] study. Since the use of an exclusive human milk-based diet with a donor human milk derived fortifier is a relatively new feeding strategy for preterm infants, we chose to compare our cohort to two cohorts that had received this diet and a cohort that received what is considered a routine diet for preterm infants. The three previous human milk fed cohorts from the Sullivan et al [2] study are listed as the following: mother’s own milk (supplemented with pasteurized donor human milk) fortified with donor human milk derived fortifier at 40 ml/kg/d (HMF 40), mother’s own milk (supplemented with pasteurized donor human milk) fortified with donor human milk derived fortifier at 100 ml/kg/d (HMF 100), and mother’s own milk fortified with bovine milk-based fortifier at 100 ml/kg/d or preterm formula if mother’s own milk unavailable (Bovine HMF or formula). The donor human milk derived fortifier used in the HMF 40 and HMF 100 cohort provided an additional 4 kcal/oz (0.14 kcal/g) which would provide an estimated 120 kcal/kg/day and 2.85 g/kg/day of protein if infants were assumed to receive mature human milk at a volume of 150 ml/kg/day. The bovine milk-based fortifiers used in the Bovine HMF cohort provided an additional 4 kcal/oz (0.14 kcal/g) and an estimated 120 kcal/kg/day with 2.9 or 3.9 g/kg/day protein depending on which bovine fortifier was used.

### Statistical analyses

Relationships among groups were evaluated using general linear modeling in which growth parameters were the primary outcomes. Paired t-test was used to compare birth and discharge percentiles of infants and regression analysis was used to compare relationships between growth velocities and change in these percentiles. Additionally, comparisons between our cohort and previous human milk fed cohorts were performed as follows: Kruskal-Wallis test for baseline quantitative outcomes, chi-squared test for baseline qualitative outcomes, Kaplan-Meier method with log rank test for median number of TPN days, and Kruskal-Wallis test for comparison of growth velocities with pairwise comparisons using Fisher’s LSD test. Statistical significance was defined as p <0.05. Analyses were completed using SPSS 19.0 (SPSS Inc., Chicago, IL). All data are mean ± standard deviation unless otherwise noted.

## Results

**Figure 1:**
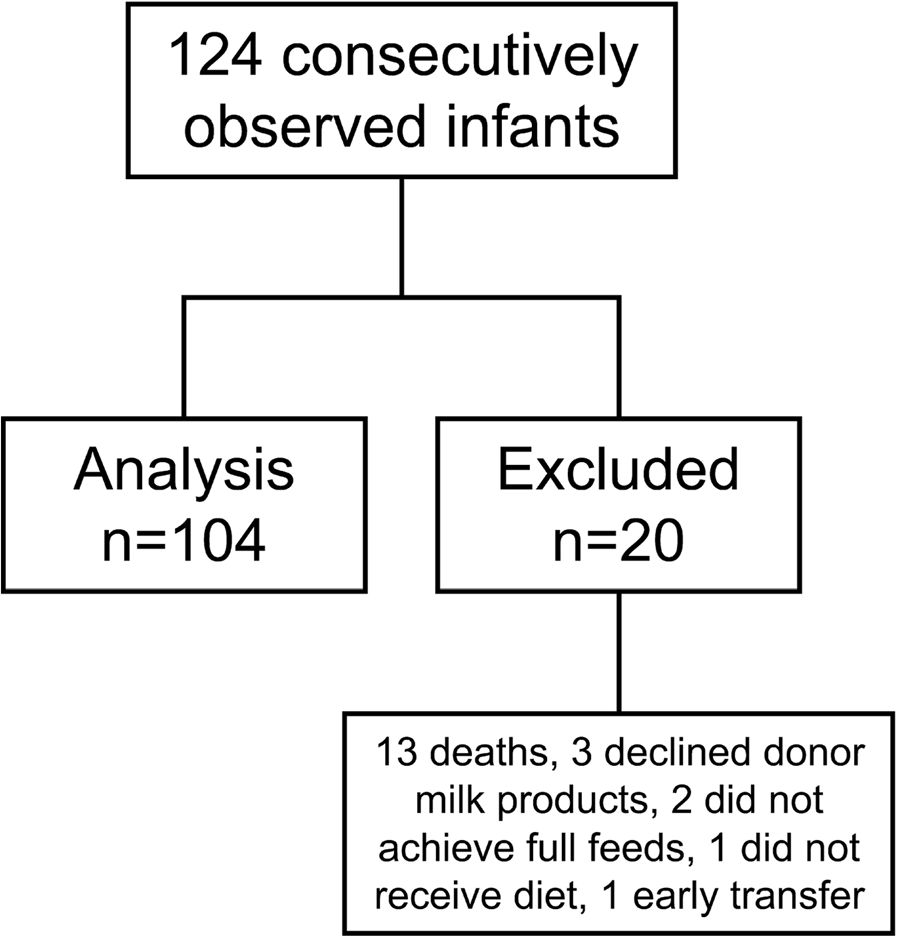
104 infants ≤ 1250 g BW receiving an exclusive human milk-based diet were prospectively followed.

**Figure 2:**
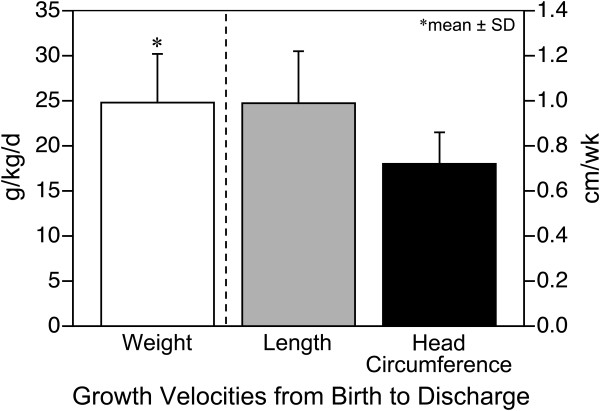
Growth velocities from birth to discharge.

We consecutively observed 104 infants (Figure 1) with demographics and study outcomes presented in Tables (Table 1) (Table 2). Mean weight gain was 24.8 ± 5.4 g/kg/d which exceeded the targeted growth standard of 15 g/kg/d (Figure 2). In the cohort, 98% of infants had a weight velocity from birth to discharge greater than the goal of 15 g/kg/day with 81% of infants achieving > 20 g/kg/day. Mean length gain was 0.99 ± 0.23 cm/wk which met the targeted standard of 1.0 cm/wk and 50% of infants had a length velocity greater than this target (Figure 2). Mean gain in HC was 0.72 ± 0.14 cm/wk which met the targeted standard and 61% of infants had a HC velocity greater than this target (Figure 2). When evaluating growth velocities at 40 weeks PMA (or before if discharged prior to 40 weeks PMA), which gave a mean PMA of 38.5 ± 1.8 weeks, mean weight gain was 24.4 ± 5.5 g/kg/day, length gain was 1.01 ± 0.2 cm/wk, and HC gain was 0.75 ± 0.14 cm/wk.

**Table 1:** Demographics for infants ≤ 1250 g BW receiving an exclusive human milk-based diet

	**n = 104**
Birth weight, g	913 ± 182^*^
Gestational age, wks	27.6 ± 2.0
Male, n (%)	49 (47)
Race: White, n (%)	28 (27)
Black	40 (38)
Hispanic	24 (23)
Other	12 (12)
Birth length, cm	34.4 ± 2.6
Birth head circumference, cm	24.2 ± 1.8
APGAR 5 minute	7 ± 2
Inborn, n (%)	59 (57)
Antenatal steroids, n (%)	77 (74)

^*^Mean ± SD.

**Table 2:** Outcomes for infants ≤ 1250 g BW receiving an exclusive human milk-based diet

		**n = 104**
Weight gain, g/kg/d		24.8 ± 5.4^*^
Length, cm/wk		0.99 ± 0.23
Head circumference, cm/wk		0.72 ± 0.14
Days to regain birth weight		8.4 ± 4.0
Days to full feeds		14 (12,19)^†^
Days to fortification of feeds		10 (8,14)
Volume at which feeds were fortified, ml/kg/d		80 (60, 90)
Parenteral nutrition days		13 (10, 19)
Transition to bovine products, wks PMA		36 ± 1.5
Necrotizing enterocolitis	Medical, n (%)	3 (3)
	Surgical, n (%)	1 (1)
Spontaneous intestinal perforation, n (%)		2 (2)
Late onset sepsis, n (%)		14 (13)
Patent ductus arteriosus, n (%)		49 (47)
No IVH, n (%)		78 (75)
IVH: Grade III or IV, n (%)		5 (5)
Bronchopulmonary dysplasia, n (%)		46 (44)
Weight at discharge, g		2795 (2247, 3155)
Length of stay, d		82 (68, 106)
Post-menstrual age at discharge, wks		40.8 ± 6.0
SGA at birth, n (%)		22 (21)
SGA at discharge or 40 weeks PMA, n (%)		45 (43)

^*^Mean ± SD, ^†^Median (25^th^, 75^th^ percentile).

Forty-nine infants (47%) received > 90% mother’s own milk. At the time of discharge home, 7 infants (7%) were receiving only human milk and 39 infants (38%) were receiving any human milk. Weight velocity from birth to discharge was significantly affected by the day of fortification of feeds (p = 0.005), days to reach full enteral feeds (p = 0.02), and BW (p < 0.001) but not gender or race. In addition, the change in percentile on the growth curve from BW to discharge weight was significantly affected by the weight gain (p = 0.04). The number of days to reach full enteral feeds was affected by the presence of a PDA (p < 0.001) as well as NEC, intestinal perforation, or ileus associated with sepsis (p < 0.001) but not by gestational age or gender. Increased days on TPN was associated with an increased risk of late onset sepsis (p < 0.001).

Although it is not routine practice at our institution, thirteen infants (13%) received additional supplementation secondary to severe growth failure: 5 received donor human milk that was 22 kcal/oz (0.73 kcal/g), 3 received expressed human milk obtained from the infant’s mother at the end of pumping, 3 received Beneprotein® (Nestle Nutrition, http://www.nestle-nutrition.com), 2 received Polycose® (Abbott Nutrition, http://abbottnutrition.com), 1 received corn oil, 1 received safflower oil, and 1 received MCT oil. These infants were mostly fluid restricted and on diuretics for significant bronchopulmonary dysplasia. Additional supplementation did not significantly affect weight gain (p = 0.23), length gain (p = 0.47) or head circumference gain (p = 0.16) from birth to discharge.

Three infants received insulin in the first week of life for hyperglycemia and 10 infants received postnatal steroids. There were 11 sets of twins in the cohort. There were an additional 13 infants that were twins but only one twin met weight criteria for the study. There was one set of triplets and 2 of 3 infants were followed. Weight gain from birth to discharge was not affected by whether the infant was a twin or multiple.

Twenty-two infants (21%) were SGA at birth and 100% of these infants were SGA at discharge or 40 weeks PMA. Of the eighty-two (79%) infants born AGA, only 23 (22%) infants were categorized as SGA at discharge or 40 weeks PMA. Overall, forty-five infants (43%) were SGA at discharge or 40 weeks PMA (mean PMA was 38.5 ± 1.8 weeks for this comparison) based on recently available intrauterine-based growth curves [18]. When comparing AGA to SGA infants, there was no difference in growth velocities for weight, length, and HC. When comparing the change in percentile on growth curves for all infants from birth to discharge, there was a significant difference for weight, length and head circumference (p < 0.001). Weight gain (p < 0.001, R^2^ = 0.21), length gain (p < 0.001, R^2^ = 0.13) and head circumference gain (p < 0.001, R^2^ = 0.08) were negatively correlated with change in percentile from birth to discharge. As gain increased, there was less difference in birth and discharge percentiles.

For the 22 infants that were SGA at birth, discharge weight percentile was affected by birth weight (p = 0.01) and birth weight percentile (p < 0.001) but not weight gain (g/kg/d) from birth to discharge, when feeds were fortified, or days to full enteral feeds. For the 82 AGA infants, discharge weight percentile was affected by birth weight (p = 0.003), birth weight percentile (p = 0.001), weight gain (g/kg/d) from birth to discharge (p = 0.010) and when feeds were fortified (p = 0.04) but not days to full enteral feeds (p = 0.32).

There were three cases of medical NEC and one surgical NEC case. All infants were AGA at birth. Two cases of medical NEC occurred at 29 and 30 weeks PMA while infants were receiving an exclusive human milk-based diet. The third case of medical NEC occurred at 37 weeks PMA while the infant was receiving all formula feeds. The case of surgical NEC occurred at 31 weeks PMA while the infant was receiving an exclusive human milk-based diet. The infant required exploratory laparotomy a week after initial diagnosis and underwent a subtotal colectomy and end-ileostomy.

Our cohort data were then compared to three previous human milk fed cohorts (Table 3). Baseline variables were similar between the 4 groups except for a significant difference between race (p < 0.001) consistent with our single site cohort compared to a multi-center study. When comparing growth rates of our cohort to previous human milk fed cohorts, our cohort had significantly greater growth in weight (p < 0.001) and length (p = 0.008) but no difference in head circumference (p = 0.84) (Table 3). Pairwise comparisons were significant with global 5% significance between the 4 groups for the following: weight gain for our cohort was significantly higher than each of the other 3 groups and the Bovine HMF or Formula group was significantly higher than HMF 100 group; length growth was significantly higher in our cohort than HMF 100 group. There were no significant pairwise differences for head circumference growth between the groups. When comparing feeding data, our cohort reached full feeds sooner, had less TPN days, and had earlier fortification of feeds (Table 3).

**Table 3:** Comparison of current study cohort and other human milk fed cohorts

	Current study human milk + HMF 60^* ^**(n = 104)**	**Human milk + HMF 40**^**† **^**(n = 71)**	**Human milk + HMF 100**^**† **^**(n = 67)**	**Bovine HMF or formula**^**† **^**(n = 69)**	**p-value**
Birth weight, g	913 ± 182^‡^	909 ± 193	945 ± 202	922 ± 197	0.67
Birth length, cm	34.4 ± 2.6	34.9 ± 3.2	35.2 ± 3.1	35.0 ± 2.9	0.16
Birth head circumference, cm	24.2 ± 1.8	24.6 ± 1.9	24.6 ± 2.1	24.7 ± 1.6	0.35
Gestational age, wks	27.6 ± 2.0	27.1 ± 2.3	27.2 ± 2.2	27.3 ± 2.0	0.70
APGAR 5 minute	7 ± 2	8 ± 1	7 ± 2	7 ± 2	0.06
Male/Female, n (%)	49/55 (47/53)	25/46 (35/65)	32/35 (48/52)	36/33 (52/48)	0.21
Race: White, n (%)	28 (27)	37 (52)	33 (49)	34 (49)	0.001
Black	40 (38)	17 (24)	20 (30)	10 (14)	
Hispanic	24 (23)	15 (21)	13 (19)	22 (32)	
Other	12 (12)	2 (3)	1 (2)	2 (3)	
Antenatal steroids, n (%)	77 (74)	51 (72)	56 (83)	53 (77)	0.39
Weight gain (g/kg/d)	24.8 ± 5.4	14.6 ± 4.5	14.0 ± 2.9	16.0 ± 7.8	<0.001
Length (cm/wk)	0.99 ± 0.23	0.93 ± 0.53	0.86 ± 0.44	0.93 ± 0.35	0.008
Head circumference (cm/wk)	0.72 ± 0.14	0.72 ± 0.22	0.71 ± 0.22	0.74 ± 0.22	0.84
Days to full feeds	18.2 ± 10.6	24.4 ± 12.7	26.5 ± 18.0	25.0 ± 13.5	<0.001
Day of life feeds initiated	3.3 ± 2.9	5.6 ± 6.6	4.3 ± 3.9	4.7 ± 4.8	0.56
Day of life feeds fortified	13.0 ± 8.3	14.1 ± 9.0	21.0 ± 14.9	18.4 ± 9.2	<0.001
TPN days	13 (10, 19)^§^	20 (11,33)	20 (13,34)	22 (13,34)	<0.001
% of human milk that was mother’s own milk	65.7 ± 40.3	70.6 ± 40.9	67.5 ± 37.2	N/A	0.046

^*^mother’s own milk or donor human milk fortified with donor human milk derived fortifier at 60 ml/kg/d, ^†^Sullivan et. al, J Peds, 2010, ^‡^Mean ± SD, ^§^Median (25^th^, 75^th^ percentile).

## Discussion

In 104 premature infants receiving a feeding protocol providing an exclusive human milk-based diet, we demonstrated that weight gain exceeded targeted growth standards and length and HC gain met targeted standards [4,9]. We speculate that the early introduction and rapid advancement of human milk fortification in our protocol led to achievement of adequate growth rates. As evidence for this, we found that weight gain from birth to discharge was significantly affected by the day of fortification of feeds and days to reach full enteral feeds. With rapid advancement of human milk fortification using a donor human milk derived fortifier, infants received an additional 6 kcal/oz (0.21 kcal/g) prior to achievement of full feeds. Once infants achieved full feeds, they received a minimum estimated caloric intake of 130 kcal/kg/d with 3.6 g/kg/d of protein but often received 140 kcal/kg/d with 4.4 g/kg/d of protein if growth did not meet targeted standards. This lead to increased caloric and protein intake as compared to other feeding strategies [2] and lead to improved growth which is supported by literature suggesting improved outcomes with early nutrition [15,21]. In addition to meeting targeted growth, infants achieved full feeds earlier and had less TPN days as compared to other human milk fed cohorts. Although not statistically significant, earlier initiation of feeds in our cohort may have contributed to improvement in growth as well. There was a low rate of NEC observed with this feeding strategy.

When comparing growth rates of our cohort to previous human milk fed cohorts, our cohort had significantly greater growth in weight and length but no difference in head circumference. This study provides data showing that infants can achieve and mostly exceed targeted growth standards when receiving an exclusive human milk-based diet. It is important to note that infants were fed differently in our cohort as stated above with early and rapid advancement of fortification of feeds. We speculate that this fortification strategy contributed to the mean weight gain of about 25 g/kg/d.

All infants who were SGA at birth remained so at discharge which is consistent with literature that there is a greater incidence of postnatal growth failure in SGA premature infants [8,22]. Discharge weight percentile for SGA infants was affected by birth weight and weight percentile at birth but not weight gain, when feeds were fortified, or days to full enteral feeds. When comparing SGA to AGA infants, there was no difference in growth velocities. This suggests that the growth of SGA infants is affected by additional factors unrelated to fortification and obtainment of full feeds as compared to AGA infant.

Despite current nutritional strategies aimed at early appropriate nutrition, a large proportion of preterm infants have extrauterine growth restriction [8,10,11,22]. Outcomes for very low birth weight infants from January 1995 to December 1996 were reported by The National Institute for Child and Human Development Neonatal Research Network for 4,438 infants weighing 501 to 1500 g at birth [22]. In this cohort, 22% of infants were SGA at birth and 97% of them had growth failure at 36 weeks corrected age [22]. For infants 501 to 1,000 g at birth, 17% were SGA at birth with 99% growth failure at 36 weeks corrected age [22]. Another large study by Clark et al., which was a database review of growth data on 24,371 premature neonates born between 23 and 34 weeks’ gestational age, found that the incidence of extrauterine growth restriction at discharge was 28% and the incidence increased with decreasing gestational age and birth weight [11].

In our study, infants’ weight gain exceeded targeted standards while receiving a feeding protocol providing early and rapid advancement of fortification of human milk; however 43% of infants had extrauterine growth restriction at discharge or 40 weeks PMA. This is an improvement from most previous literature, but further improvement is needed as growth during the postnatal period is critical. Ehrenkranz et al. reported that as the rate of weight gain and HC increased in extremely low birth weight infants, the incidence of cerebral palsy and poor neurodevelopmental scores decreased significantly at 18 to 22 months’ corrected age [15].

One study of infants < 28 weeks gestation showed that although infants met the current standard weight gain of 15 g/kg/d, infants still had extrauterine growth restriction [12]. Our cohort had a mean weight gain of 24.8 g/kg/d with 98% of infants achieving a weight gain greater than 15 g/kg/day and despite this, 43% had postnatal growth failure. We evaluated percentiles for weight, length, and head circumference at discharge or 40 weeks PMA as infants were plotted and compared to growth curves published by Olsen et al [18]. We used these growth curves for comparison because they are intrauterine growth curves based on large and racially diverse United States birth data from 22 to 42 weeks gestational age [18]. When excluding infants born SGA in the cohort, the rate of AGA infants with a weight < 10^th^ percentile at discharge or 40 weeks PMA was 22%. When comparing the change in percentile from birth to discharge for all infants, there was a significant difference for weight, length and head circumference. As growth increased, there was less difference in birth and discharge percentiles suggesting that improved growth helps maintain growth percentile as reported in the literature [12].

One study limitation was that by design, we only evaluated those infants who were able to achieve full feeds and therefore excluded a small number of infants. Two infants did not achieve full feeds by 3 months of age. Of the 13 infants that died, 9 (69%) died within the first two week of age. Although this exclusion may have led to a study population that may have had less severity of illness, the majority of infants were excluded (14 infants) because they died within the first two weeks of age or did not receive the feeding protocol. One of the strengths of this study was that infants were fed a standardized feeding protocol as tolerated using the same defined diet of an exclusive human milk-based diet with a donor human milk derived fortifier.

## Conclusions

A feeding protocol for infants ≤ 1250 g BW providing an exclusive human milk-based diet with early and rapid advancement of fortification was associated with weight gain exceeding targeted standards and with length and HC growth meeting targeted standards. Infants had a lower rate of extrauterine growth restriction compared to historical literature but further improvement is needed. Consistent nutritional policies using this approach may be considered for this population.

## Abbreviations

AGA: Appropriate for gestational age; cm/wk: Centimeters per week; g: Grams; BW: Birth weight; g/kg/d: Grams per kilogram per day; HC: Head circumference; IVH: Intraventricular hemorrhage; kcal/oz: Kilocalories per ounce; kcal/g: Kilocalories per gram; kcal/kg/day: Kilocalories per kilogram per day; mL/kg/d: Milliliters per kilogram per day; NEC: Necrotizing enterocolitis; PMA: Postmenstrual age; SGA: Small for gestational age; TPN: Total parenteral nutrition; VLBW: Very low birth weight.

## Competing interests

The authors declare that they have no competing interests.

## Authors’ contributions

AH designed the study, carried out acquisition and analysis of the data, supervised data collection, interpreted the data, drafted the manuscript, and approved the final manuscript as submitted. KH designed the study, carried out data acquisition, supervised data collection, interpreted the data, participated in writing and editing the manuscript, and approved the final manuscript as submitted. KC carried out data acquisition, interpreted the data, participated in writing and editing the manuscript, and approved the final manuscript as submitted. SA designed the study, supervised data collection, carried out analysis of the data, interpreted the data, participated in writing and editing the manuscript, and approved the final manuscript as submitted.

## References

[B1] Schanler RJ Outcomes of human milk-fed premature infants Semin Perinatol 2011 6 1 29 33 10.1053/j.semperi.2010.10.005 21255704

[B2] Sullivan S Schanler RJ Kim JH Patel AL Trawoger R Kiechl- Kohlendorfer U An exclusively human milk-based diet is associated with a lower rate of necrotizing enterocolitis than a diet of human milk and bovine milk-based products J Pediatr 2010 6 4 562 567 10.1016/j.jpeds.2009.10.040 20036378

[B3] Quigley M Henderson G Anthony MY McGuire W Formula milk versus donor breast milk for feeding preterm or low birth weight infants Cochrane Database of Syst Rev 2007 6 CD002971 10.1002/14651858.CD002971.pub2 17943776

[B4] Lucas A Gore SM Cole TJ Bamford MF Dossetor JF Barr I Multicentre trial on feeding low birth weight infants: effects of diet on early growth Arch Dis Child 1984 6 8 722 730 10.1136/adc.59.8.722 6476868 PMC1628628

[B5] American Academy of Pediatrics Section on Breastfeeding Breastfeeding and the use of human milk Pediatrics 2012 6 3 e827 e841 22371471 10.1542/peds.2011-3552

[B6] Hawthorne KM Griffin IJ Abrams SA Current issues in nutritional management of very low birth weight infants Minerva Pediatr 2004 6 4 359 372 15457134

[B7] Morales Y Schanler RJ Human milk and clinical outcomes in VLBW infants: how compelling is the evidence of benefit? Semin Perinatol 2007 6 2 83 88 10.1053/j.semperi.2007.02.002 17462492

[B8] Dusick AM Poindexter BB Ehrenkranz RA Lemons JA Growth failure in the preterm infant: can we catch up? Semin Perinatol 2003 6 4 302 310 10.1016/S0146-0005(03)00044-2 14510321

[B9] Lubchenco LO Hansman C Boyd E Intrauterine growth in length and head circumference as estimated from live births at gestational ages from 26 to 42 weeks Pediatrics 1966 6 3 403 408 5906365

[B10] Ehrenkranz RA Younes N Lemons JA Fanaroff AA Donovan EF Wright LL Longitudinal growth of hospitalized very low birth weight infants Pediatrics 1999 6 2 pt 1 280 289 10429008 10.1542/peds.104.2.280

[B11] Clark RH Thomas P Peabody J Extrauterine growth restriction remains a serious problem in prematurely born neonates Pediatrics 2003 6 5 pt 1 986 990 12728076 10.1542/peds.111.5.986

[B12] Martin CR Brown YF Ehrenkranz RA O’Shea TM Allred EN Belfort MB Nutritional practices and growth velocity in the first month of life in extremely premature infants Pediatrics 2009 6 2 649 657 10.1542/peds.2008-3258 19651583 PMC2859427

[B13] Tsang RC Uauy R Koletzko B Zlotkin SH Nutrition of the preterm infant: scientific basis and practical guidelines 2005 Cincinnati, OH: Digital Educational Publishing Inc 415 416

[B14] Agostoni C Buonocore G Carnielli VP De Curtis M Darmaun D Decsi T Enteral nutrient supply for preterm infants: commentary for the European society for pediatric gastroenterology, hepatology, and nutrition committee on nutrition JPGN 2010 6 1 85 91 19881390 10.1097/MPG.0b013e3181adaee0

[B15] Ehrenkranz RA Dusick AM Vohr BR Wright LL Wrage LA Poole WK Growth in the neonatal intensive care unit influences neurodevelopmental and growth outcomes of extremely low birth weight infants Pediatrics 2006 6 4 1253 1261 10.1542/peds.2005-1368 16585322

[B16] Prolacta Bioscience, Industry, California: product descriptionhttp://www.prolacta.com/human-milk-fortifier

[B17] American Academy of Pediatrics: Committee on Nutrition. Nutritional needs of preterm infants Kleinman RE Nutritional needs of preterm infants Pediatric nutrition handbook: 6th edition 2009 Elk Grove, IL: American Academy of Pediatrics 79 112

[B18] Olsen IE Groveman SA Lawson ML Clark RH Zemel BS New intrauterine growth curves based on United States data Pediatrics 2010 6 2 e214 e224 10.1542/peds.2009-0913 20100760

[B19] Walsh MC Kliegman RM Necrotizing enterocolitis: treatment based on staging criteria Pediatr Clin North Am 1986 6 1 179 201 3081865 10.1016/S0031-3955(16)34975-6 PMC7131118

[B20] Papile L-A Burstein J Burstein R Koffler H Incidence and evolution of subependymal and intraventricular hemorrhage: a study of infants with birth weights less than 1,500 gm J Pediatr 1978 6 4 529 534 10.1016/S0022-3476(78)80282-0 305471

[B21] Ehrenkranz RA Early nutritional support and outcomes in ELBW infants Early Hum Dev 2010 6 1 21 25 10.1016/j.earlhumdev.2010.01.014 20123153

[B22] Lemons JA Bauer CR Oh W Korones SB Papile LA Stoll BJ Very low birth weight outcomes of the national institute of child health and human development neonatal research network, january 1995 through december 1996 Pediatrics 2001 6 1 E1 10.1542/peds.107.1.e1 11134465

